# Horizontal Transfer of Different *erm*(B)-Carrying Mobile Elements Among *Streptococcus suis* Strains With Different Serotypes

**DOI:** 10.3389/fmicb.2021.628740

**Published:** 2021-03-26

**Authors:** Li Chen, Jinhu Huang, Xinxin Huang, Yuping He, Junjie Sun, Xingyang Dai, Xiaoming Wang, Muhammad Shafiq, Liping Wang

**Affiliations:** ^1^Ministry of Education (MOE) Joint International Research Laboratory of Animal Health and Food Safety, College of Veterinary Medicine, Nanjing Agricultural University, Nanjing, China; ^2^Technical Center for Animal, Plant and Food Inspection and Quarantine of Shanghai Customs, Shanghai, China

**Keywords:** *erm*(B), ICEs, GIs, horizontal transfer, *S. suis*

## Abstract

Macrolide-resistant *Streptococcus suis* is highly prevalent worldwide. The acquisition of the *erm*(B) gene mediated by mobile genetic elements (MGEs) in particular integrative and conjugative elements (ICEs) is recognized as the main reason for the rapid spread of macrolide-resistant streptococcal strains. However, knowledge about different *erm*(B)-carrying elements responsible for the widespread of macrolide resistance and their transferability in *S. suis* remains poorly understood. In the present study, two *erm*(B)- and *tet*(O)-harboring putative ICEs, designated as ICE*Ssu*YSB17_*rplL* and ICE*Ssu*YSJ15_*rplL*, and a novel *erm*(B)- and *aadE-spw*-like-carrying genomic island (GI), named GI*Ssu*JHJ17_*rpsI*, were identified to be excised from the chromosome and transferred among *S. suis* strains with different serotypes. ICE*Ssu*YSB17_*rplL* and ICE*Ssu*YSJ15_*rplL* were integrated downstream the *rplL* gene, a conserve locus of the ICE*Sa*2603 family. GI*Ssu*JHJ17_*rpsI*, with no genes belonging to the conjugation module, was integrated into the site of *rpsI*. All transconjugants did not exhibit obvious fitness cost by growth curve and competition assays when compared with the recipient. The results demonstrate that different *erm*(B)-carrying elements were presented and highlight the role of these elements in the dissemination of macrolide resistance in *S. suis*.

## Introduction

The rapid increase of macrolide resistance in *Streptococcus* has been reported worldwide from both pig and human isolates during the past two decades (Princivalli et al., 2009; Palmieri et al., 2011; Vela et al., 2017). Although numerous resistance genes have been reported since the early 1980’s^1^ (Roberts, 2008), macrolide resistance in streptococci is primarily due to the ribosomal alteration of the 23S rRNA target site by methylases encoded by the *erm* genes, predominantly *erm*(B), which mediate resistance to macrolides, lincosamides, and streptogramin B (MLS_*B*_) antimicrobials, and active efflux by the *mef* and *msr* genes (Wilson, 2014; Fyfe et al., 2016). These resistance genes are frequently carried by mobile genetic elements (MGEs), such as plasmids, transposons, prophages, and more recently, integrative and conjugative elements (ICEs) (Horaud et al., 1985; Woodbury et al., 2008; Varaldo et al., 2009; Huang et al., 2016b,c; Feßler et al., 2018; Libante et al., 2019). ICEs primarily reside in the bacterial chromosome and can excise from the donor chromosome to form a circular molecule that can be horizontally self-transferred to a recipient cell by conjugation (Bellanger et al., 2014). Other chromosomal elements, including integrative and mobilizable elements (IMEs), which encode a recombinase and only some conjugation proteins, and some genomic islands (GIs), which encode a recombinase but do not encode any conjugation proteins, were recently found to be mobilized in *trans* by ICEs (Daccord et al., 2010) and might have played crucial roles in bacterial evolution.

The *erm*(B) gene was originally identified on a 5,266 bp transposon Tn*917* from *Enterococcus faecalis* (Tomich et al., 1979). In human streptococci strains, the *erm*(B)-containing Tn*917* was usually integrated into Tn*916* (designated as Tn*3872*), which also carries the tetracycline resistance gene *tet*(M) (Brenciani et al., 2007; Varaldo et al., 2009). Further, two other *erm*(B)-containing elements, *erm*(B) element and macrolide–aminoglycoside–streptothricin element, were frequently inserted into *tet*(M)-carrying Tn*916*-like structure (e.g., Tn*6002*/Tn*6003*, Tn*1545*, Tn*2009*/Tn*2010*) (Varaldo et al., 2009; Marosevic et al., 2017). This genetic linkage between *erm*(B) and *tet*(M) on different MGEs was considered to be the primary mechanism for the spread of streptococcal bacteria that are resistant to both macrolide and tetracycline antimicrobials (Brenciani et al., 2007; Cochetti et al., 2008; Xu et al., 2010). However, in the zoonotic pathogen *Streptococcus suis*, the linkage between *erm*(B) and *tet*(O) was more frequently detected in different countries (Martel et al., 2005; Gurung et al., 2015; Huang et al., 2015; Bojarska et al., 2016; Pan et al., 2019), suggesting that MGEs responsible for macrolide and tetracycline resistance might be different from other streptococci (Huang et al., 2016b,c). *S. suis* is a key antibiotic resistance gene reservoir and a major zoonotic pathogen responsible for severe economic loss to the swine industry. This bacterium causes specific diseases in humans after contact with infected animals or derived food products. It caused human infection outbreaks in China in 1998 and 2005, respectively, and sporadic cases of *S. suis* infections in humans have occurred occasionally worldwide (Hui et al., 2005; Mazokopakis et al., 2005; Yu et al., 2006; Mai et al., 2008; CDC, 2013; Huang et al., 2019). Recent studies have demonstrated that the *erm*(B) and *tet*(O) genes co-existed on different ICEs in *S. suis* isolates of both pig and human origins (Holden et al., 2009; Zhang et al., 2011; Huang et al., 2016a,c). Previous results from our laboratory and other investigators have confirmed the intra-species transfer of the *erm*(B)- and *tet*(O)-carrying ICEs by conjugation (Huang et al., 2016a,c; Zhou et al., 2017; Pan et al., 2019). However, knowledge about types of *erm*(B) elements responsible for widespread macrolide resistance remains rare. In the present study, we identified three *erm*(B)-carrying transferable elements, including two *erm*(B)- and *tet*(O)-harboring putative ICEs, belonging to the ICE*Sa*2603 family, and a novel *erm*(B)-carrying GI, which can be horizontally transferred among *S. suis* strains with different serotypes.

## Materials and Methods

### Bacterial Strains and Culture Condition

In this study, a total of 320 *S. suis* isolates obtained from humans and pigs in China from 2005 to 2018 were included. All *S. suis* strains were routinely cultivated on Todd–Hewitt broth (THB) or Todd–Hewitt agar (THA) plates supplemented with 5% calf serum at 37°C.

### Genomic DNA Extraction and PCR Amplification

The crude genomic DNA was prepared using boiling extraction. The bacterial cultures were centrifuged (6,000*g* for 5 min at room temperature), and the pellets were harvested and resuspended in TE buffer (10 mM Tris–HCl, 1 mM EDTA, pH = 8.0). The mixtures were boiled for 10 min and incubated with ice for 10 min, then the mixtures were centrifuged, and the supernatants were collected. The extracted DNA was used as the template for PCR. All *S. suis* isolates were subjected to screen for the resistance genes of *erm*(B) and *tet*(O) in PCR analysis. The ICE*Sa*2603 family conserved genes of *Int*_tyr_ and *virB4* were characterized by a PCR mapping assay. To investigate the presence of circular/integrate forms of ICE and GI, two specific primer pairs (P1–P4 for ICE*Ssu*YSB17_*rplL* and P5–P8 for GI*Ssu*JHJ17_*rpsI*) were designed and used in PCR experiments. All the PCR primers were listed in Supplementary Table S1. Amplification reactions were performed in a total volume of 25 μl containing 12.5 μl 2 × Taq Plus Master Mix II (Vazyme, China), 1 μl of each primer (10 μM), 1 μl genomic DNA, and 9.5 μl water. The PCR assay was carried out in a thermocycler, comprising 5 min of pre-incubation at 94°C, followed by 35 cycles of 30 s at 94°C, 30 s at 50–60°C (determined by primers), and 1 min at 72°C. The final extension was performed for 10 min at 72°C.

### Antibiotic Susceptibility Testing

Antimicrobial susceptibility testing was performed for determining the minimum inhibitory concentrations (MICs) to the corresponding antimicrobial agents according to the CLSI M100-ED28 guideline (CLSI, 2018). *Staphylococcus aureus* ATCC 29213 was used for quality control.

### Transfer and Retransfer Experiments

We randomly selected six non-serotype 2 *S. suis* strains carrying the *erm*(B), *tet*(O), *virB4*, and *Int*_tyr_ genes that were used as donors (rifampicin and fusidic acid susceptibility and erythromycin resistance) (Supplementary Table S2). *S. suis* P1/7RF (rifampicin and fusidic acid resistance and erythromycin susceptibility) described in a previous study (Huang et al., 2016a) was utilized as recipients, which was considered to be not competent until the *com*RS system was activated. For a long time, *S. suis* was thought to be a bacterium unable to transformation. However, recently, the natural competence of *S. suis* under laboratory conditions was demonstrated with the addition of a *com*X-inducing peptide (Zaccaria et al., 2014). *S. suis* SH28CIP and NP4CIP (ciprofloxacin resistance and erythromycin susceptibility) were used as recipients in retransfer experiments. Transfer and retransfer experiments were performed by filter mating as described previously (Li et al., 2011; Huang et al., 2016b), with minor modifications. In brief, donor and recipient strains were grown separately at 37°C. The bacterial cultures were centrifuged to harvest at the end of the exponential growth phase and then mixed at a ratio of 1:10 (donor to recipient). The mixtures were placed on sterile nitrocellulose filters on THA plates and incubated at 37°C for 4 h. Bacteria were removed from the filters by washing with 2 ml THB medium. Transconjugants were selected by THA plates containing appropriate antibiotics (50 mg/l erythromycin with 100 mg/l rifampicin and 100 mg/l fusidic acid in transfer assays or 100 mg/l ciprofloxacin in retransfer assays) and further confirmed the presence of the *erm*(B), *tet*(O), and type IV secretion system (T4SS) core genes by PCR. To rule out spontaneous mutation and the contribution of transformation to the genetic exchange during transfer, filter mating experiments were carried out in the presence of 10 μg/ml DNase I in transfer and retransfer assays, with donor and recipient control plates included. The residual DNA with the treatment of DNase I was quantified by quantitative PCR (qPCR) using primers targeting the *virB4* gene in wash buffer. The conjugation experiments were done in triplicate. The transfer frequency was calculated based on the number of observed transconjugants divided by the donors’ initial number.

### PFGE and DNA Hybridization

To determine the location of the *erm*(B) or *tet*(O) genes, genomic DNA from each of the donor strains, the recipient strains, and the corresponding transconjugants was digested with *Sma*I endonuclease and subjected to pulsed-field gel electrophoresis (PFGE) as previously described (Vela et al., 2003; Huang et al., 2015), followed by southern blotting and DNA hybridization analysis using *erm*(B)- or *tet*(O) probes with specific primers (Supplementary Table S1).

### Whole-Genome Sequencing and Analysis

Bacterial cells were centrifuged, and the pellets were harvested and resuspended in TE buffer (10 mM Tris–HCl, 1 mM EDTA, pH 8.0). Total genomic DNA was extracted using an Omega Bacteria DNA Kit (OMEGA, China) according to the manufacturer’s instructions. Purified genomic DNAs were submitted for 150 bp paired-end whole-genome sequencing (WGS) on the Illumina Hiseq 2000 platform (Biozeron, Shanghai, China). ABySS v2.0.2 was used for genome assembly with multiple-Kmer parameters (Jackman et al., 2017). The genomes were annotated using the Rapid Annotation of microbial genomes using Subsystems Technology (RAST) annotation server^2^ (Overbeek et al., 2014), and the genetic elements were predicted using the ICEfinder^3^. ICEs and GI were identified by comparison with other MGEs from GenBank and were visualized using Mauve and Easyfig 2.2.2 (Sullivan et al., 2011).

### Growth Curve and Fitness Measurements

The fitness difference between transconjugants and the recipient strains was calculated by *in vitro* growth and competition assays as described previously (Gagneux et al., 2006; Kodio et al., 2019). In *in vitro* growth assay, a single colony of each strain was picked from the agar plate and incubated overnight at 37°C Cultures were adjusted into the same optical density (OD), diluted 1:100 in fresh THB medium, and aliquoted to 1 ml at an interval of every hour, and the OD600 of bacterial cultures was measured for 24 h.

In *in vitro* competition assay, cultures of each competitor were adjusted to OD_600_ = 0.1, mixed in a 1:1 ratio, and diluted to 1:100 in 10 ml at 37°C for 24 h. The mixtures at both startpoint (0 h) and endpoint (24 h) were plated on THA plates without or with 50 mg/l erythromycin and incubated at 37°C for 48 h. The relative competitive fitness W was calculated using the formula W = ln(R_f_/R_i_)/ln(S_f_/S_i_). R_i_ and S_i_ indicate the number of transconjugant and recipient cells at 0 h, respectively, and R_f_ and S_f_ indicate the number of transconjugant and recipient cells at 24 h, respectively.

### GenBank Accession Numbers

The complete nucleotide sequences of ICE*Ssu*YSB17_*rplL* and GI*Ssu*JHJ17_*rpsI* have been deposited in the GenBank database under accession numbers MN876247 and MN876248, respectively.

## Results

### Co-transfer of *erm*(B) With Other Antimicrobial Resistance Genes

There is a strong association between *erm*(B) and *tet*(O) in *S. suis* isolated from China and worldwide (Huang et al., 2015, 2016c). In this study, 221 *S. suis* strains (86.33%, 221/320) were co-existed of *erm*(B) and *tet*(O). In order to test the co-transfer frequency of *erm*(B) with *tet*(O), we randomly selected six *S. suis* strains as donors for conjugative transfer, which were all co-harboring the *erm*(B), *tet*(O), and T4SS core genes (Supplementary Table S2). Transconjugants were observed from strains YSB17, YSJ15, and JHJ17 under erythromycin selection with or without DNase I treatment. The residual DNA with the treatment of DNase I in mating experiments was detected by qPCR using primers targeting the *virB4* gene in wash buffer but with a negative result. For each strain, about 30–50 transconjugant clones were picked and detected to be positive for the *erm*(B), *tet*(O), and T4SS core genes by PCR. Retransfer assays using *S. suis* SH28CIP and NP4CIP as recipients were performed, but no transconjugant was obtained.

Two strains, YSB17 and YSJ15, successfully transferred the erythromycin and tetracycline resistance to recipient *S. suis* P1/7RF, with a calculated transfer frequency of (5.75 ± 1.18) × 10^–8^ and (3.84 ± 1.29) × 10^–8^. The two transconjugants, designated as SScYSB17 and SScYSJ15, respectively, exhibited macrolide and tetracycline resistance phenotypes and were tested positive for *erm*(B) and *tet*(O) (Table 1). The transfer frequency of the third transconjugant SScJHJ17 was (4.31 ± 1.53) × 10^–8^, and SScJHJ17 showed erythromycin, streptomycin, and spectinomycin resistance but tetracycline-sensitive phenotype. It acquired not only *erm*(B) and *aadE* from donor strain JHJ17, which is responsible for erythromycin and high-level streptomycin resistance, respectively, but also *spw-like*, which exhibited 96.58% identity to *spw* in *E. faecalis* strain E211 (MK784777) (Wendlandt et al., 2013), and might mediate resistance to spectinomycin in *S. suis* SScJHJ17. However, *tet*(O) carried by donor strain JHJ17 was not detected in this conjugant strain.

**TABLE 1:** Characteristics of strains included in the filter mating conjugation experiments performed in this study.

Strains	Conjugation frequency^a^	MIC (mg/l)					
		
		RIF	FUS	ERY	TET	STR	SPC
P1/7RF		**256**	**256**	0.125	0.25	1,024	32
YSB17		≤0.0625	32	**>256**	**64**	>2,048	8
SScYSB17	(5.75 ± 1.18) × 10^–8^	**256**	**256**	**>256**	**64**	1,024	32
YSJ15		≤0.0625	32	**>256**	**256**	>2,048	8
SScYSJ15	(3.84 ± 1.29) × 10^–8^	**256**	**256**	**>256**	**128**	1,024	32
JHJ17		≤0.0625	32	**>256**	32	**>2,048**	**256**
SScJHJ17	(4.31 ± 1.53) × 10^–8^	**256**	**256**	**>256**	0.25	**>2,048**	**256**

*RIF, rifampin; FUS, fusidic acid; ERY, erythromycin; TET, tetracycline; STR, streptomycin; SPC, spectinomycin. ^a^The frequency is calculated by CFUs of transconjugants/donors. Resistance-related phenotypes related to transfer in conjugation assays were shown in bold.*

Following PFGE separation, southern blotting, and hybridization with the *erm*(B) or *tet*(O) probes, the sizes of the transferable DNA fragments were deduced by comparing the profiles of the donor strains, the recipient strains, and the transconjugants. *Sma*I-PFGE analysis of YSB17 and JHJ17 conjugation pairs showed differences in three bands between the recipients and transconjugants (Supplementary Figure S1). An ∼460 kb fragment existed in the recipient P1/7RF but could not be detected in transconjugant SScYSB17. Instead, two fragments, with the sizes of ∼390 and ∼140 kb, were present in SScYSB17. These results suggested the successfully transferred element with an estimated size of approximately 70 kb, most probably ICE that carried *erm*(B) and *tet*(O), into the recipient’s genome. Subsequent DNA hybridization revealed that the genes *erm*(B) and *tet*(O) were located on the different fragments, indicating the presence of *Sma*I restriction sites within this element (Supplementary Figure S1A). Similarly, the maximal fragment of recipient P1/7RF was replaced with two smaller fragments of transconjugant SScJHJ17, and the *erm*(B) gene was located in one of the fragments that differed from the recipient P1/7RF (Supplementary Figure S1B).

### Characterization of Two *erm*(B)- and *tet*(O)-Carrying ICEs and an *erm*(B)-Carrying GI

**FIGURE 1:**
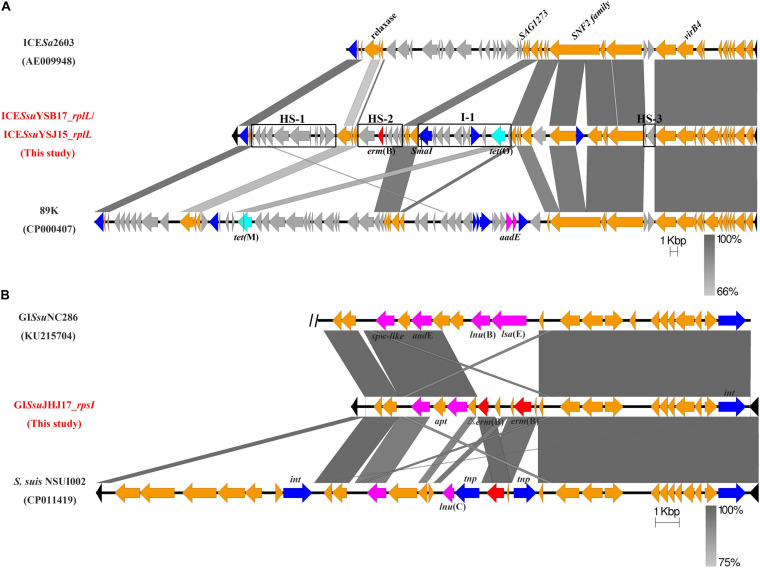
Genetic features of the mobile genetic elements ICE*Ssu*YSB17_*rplL* and GI*Ssu*JHJ17_*rpsI*. The direction of the arrow indicates the direction of transcription. Homologous regions are shaded in gray. Genes are shown in different colors: the ICE and GI flanking chromosomal genes were shown in black, the 30 conserved core genes of the ICE*Sa*2603 family backbone and GI*Ssu*JHJ17_*rpsI* backbone are in orange, and variable genes are in light gray. Integrase/transposase/recombinase genes were highlighted in blue, *erm*(B) is marked in red, tet-resistant genes are in pale blue, and other resistant genes are labeled in pink. **(A)** Comparison of ICE*Ssu*YSB17_*rplL*/ICE*Ssu*YSJ15_*rplL* with ICE*Sa*2603 and 89K. Intergenic hotspots HS-1, HS-2, and HS-3 and insertion site I-2 were indicated. *Sma*I restriction site is marked by a black arrow. **(B)** GI*Ssu*JHJ17_*rpsI* from *S. suis* JHJ17 and linear DNA comparison against part of ICE*Ssu*NC286 and fragments of *S. suis* NSUI002. The two vertical black diagonal lines on the left of NC286 indicate that there is still a part of the sequence that is not shown.

To better understand the genetic context of the *erm*(B)-carrying elements, we determined the whole genomes of the donors YSB17, YSJ15, and JHJ17 and their respective transconjugants by WGS. In both YSB17 and its transconjugant SScYSB17, a single putative ICE carrying *erm*(B) and *tet*(O) in the chromosome was identified using the ICEfinder and designated as ICE*Ssu*YSB17_*rplL*. The *hyd* and *rplL* (Figure 1A, black color) are located at the terminals of ICE*Ssu*YSB17_*rplL*, which encoded a predicted hydrolase and 50S ribosomal protein L7/L12, respectively. ICE*Ssu*YSB17_*rplL* is 69,442 bp in length, with an average G + C content of 38%, and consists of 68 putative open reading frames (ORFs). A *Sma*I site existed at 4,843 bp downstream of the *erm*(B) gene and 9,665 bp upstream of the *tet*(O) gene, which is consistent with the *Sma*I-PFGE and hybridization results (Figure 1A). A 15-bp conserved sequence (5’-TTATTTAAGAGTAAC-3’) was presented at both the left (L) and right (R) ends of the integrated ICE*Ssu*YSB17_*rplL* element. ICE*Ssu*YSB17_*rplL* was integrated into the 3’-end of the *rplL* gene and contained all 30 conserved core genes compared with ICE*Sa*2603. In addition, three intergenic hotspots (HS-1, HS-2, and HS-3) and three additional insertion sites were presented (Figure 1A). Among the three insertions, one was inserted in a previously identified site I-1, and one reverse transcriptase gene and one integrase gene were integrated within the SNF2 protein gene sequence, whereas *erm*(B) and *tet*(O) are located in HS-2 and I-1 variable regions, respectively. To trace the derivation of the resistance genes, comparative genome analyses were performed for HS-2 and I-1. The 5,869 bp HS-2 segment shared higher similarity with the corresponding sequences of the *S. suis* 9401240 (LR738724), ICE*Ssu*32457 (FR823304), ICE*Ssu*YS108 (MK211815), and *S. suis* (MN437484) (Supplementary Figure S2A). The content of 10,906-bp I-1 segment showed identical nucleotide sequence with the *S. suis* NSUI060 (CP012911), *Blautia hansenii* DSM 20583 (CP022413), *Enterocloster clostridioformis* FDAARGOS_739 (CP050964), *Enterococcus cecorum* NCTC12421 (LS483306), *Streptococcus pyogenes* NCTC12057 (LS483331), and *Eubacterium hallii* EH1 (LT907978). The only difference is that the I-1 region contained an additional ORF (1,503 bp) encoding IS4 family transposase (Supplementary Figure S2B). Comparison of ICE*Ssu*YSB17_*rplL* with some other *erm*(B)- and *tet*(O)-carrying ICEs revealed highly conserved core genes but differed greatly in non-conserved regions (Supplementary Figure S3). In YSJ15 and its transconjugant SScYSJ15, we also detected a putative ICE, designated as ICE*Ssu*YSJ15_*rplL*, neatly identical to ICE*Ssu*YSB17_*rplL* with only five nucleotide differences. In JHJ17 but not the transconjugant SScJHJ17, a *tet*(O)-carrying putative ICE with all conserved modules was integrated into the 3’-end of the *rplL* gene (data not shown).

In both JHJ17 and its transconjugant SScJHJ17, a 16,195-bp sequence with 34% GC content was considered a putative GI and designated as GI*Ssu*JHJ17_*rpsI*. GI*Ssu*JHJ17_*rpsI* carried the *erm*(B) gene and was found to be integrated into a locus *rpsI*, the 3’-end of the gene encoding the ribosomal protein S9. Apart from a gene coding an integrase, no other putative conjugative elements, such as coupling proteins or elements participating in T4SS, were observed in GI*Ssu*JHJ17_*rpsI*. GI*Ssu*JHJ17_*rpsI* encodes 22 putative ORFs, 19 of them with the same direction of transcription as that of *erm*(B). An 8-bp conserved direct repeat sequence (5’-CCTGGTTT-3’) was detected at both flanking of the GI*Ssu*JHJ17. BLAST analysis of GI*Ssu*JHJ17_*rpsI* showed that it had the highest similarity to GI*Ssu*NC286 (KU215704) and the genomic sequence of *S. suis* NSUI002 (CP011419) (Figure 1B). In addition to the *erm*(B) gene (two copies), GI*Ssu*JHJ17_*rpsI* also contained the high-level streptomycin resistance gene *aadE* and the spectinomycin resistance gene *spw-like*. These genes are in agreement with the resistance profile of JHJ17 (Table 1).

### Detection of the Extrachromosomal Circular Intermediate Forms of ICE*Ssu*YSB17_*rplL* and GI*Ssu*JHJ17_*rpsI*

ICEs and GIs can be excised from the chromosome with the aid of the integrase to generate the extrachromosomal circular form, which is the first step of its transfer lifecycle. In this study, two specific primer pairs (P1–P4 for ICE*Ssu*YSB17_*rplL* and P5–P8 for GI*Ssu*JHJ17_*rpsI*, the location of the primers were shown in Supplementary Figures S4A,B, respectively), were designed to detect the integrated and the extrachromosomal circular forms of ICE*Ssu*YSB17_*rplL* and GI*Ssu*JHJ17_*rpsI* (Supplementary Table S1). More specifically, P1/P2 and P3/P4 amplify the integrated form of ICE*Ssu*YSB17_*rplL* left and right terminals, respectively. P2/P3 detects whether there is a circular form of ICE*Ssu*YSB17_*rplL*. After ICE*Ssu*YSB17_*rplL* excision, P1/P4 detects an empty *att* site. For GI*Ssu*JHJ17_*rpsI* identification, the pairs used for P5–P8 are analogous to P1–P4. The results confirmed the presence of both the integrated and the extrachromosomal circular forms of ICE*Ssu*YSB17_*rplL* and GI*Ssu*JHJ17_*rpsI* in the original donors and the transconjugants, but absent in the recipient strain *S. suis* P1/7RF (Supplementary Figures S4C,D). The relatively low probability of occurrence of an excised form of ICE*Ssu*YSB17_*rplL* and GI*Ssu*JHJ17_*rpsI*, as reflected by the shallow bands of P2/P3 and P6/P7 PCR amplification, might be one of the causes of low frequency for transfer of these genetic elements. Analysis of the *att*ICE/*att*B and *att*L/*att*R amplicon sequences identified the 15-bp identical sequence (5’-TTATTTAAGAGTAAC-3’). Both the circular and excised forms of GI*Ssu*JHJ17_*rpsI* contained a copy of the 8-bp conserved sequence corresponding to the direct repeat sequence (5’-CCTGGTTT-3’) site (data not shown).

### Fitness of SScYSB17 and SScJHJ17

**FIGURE 2:**
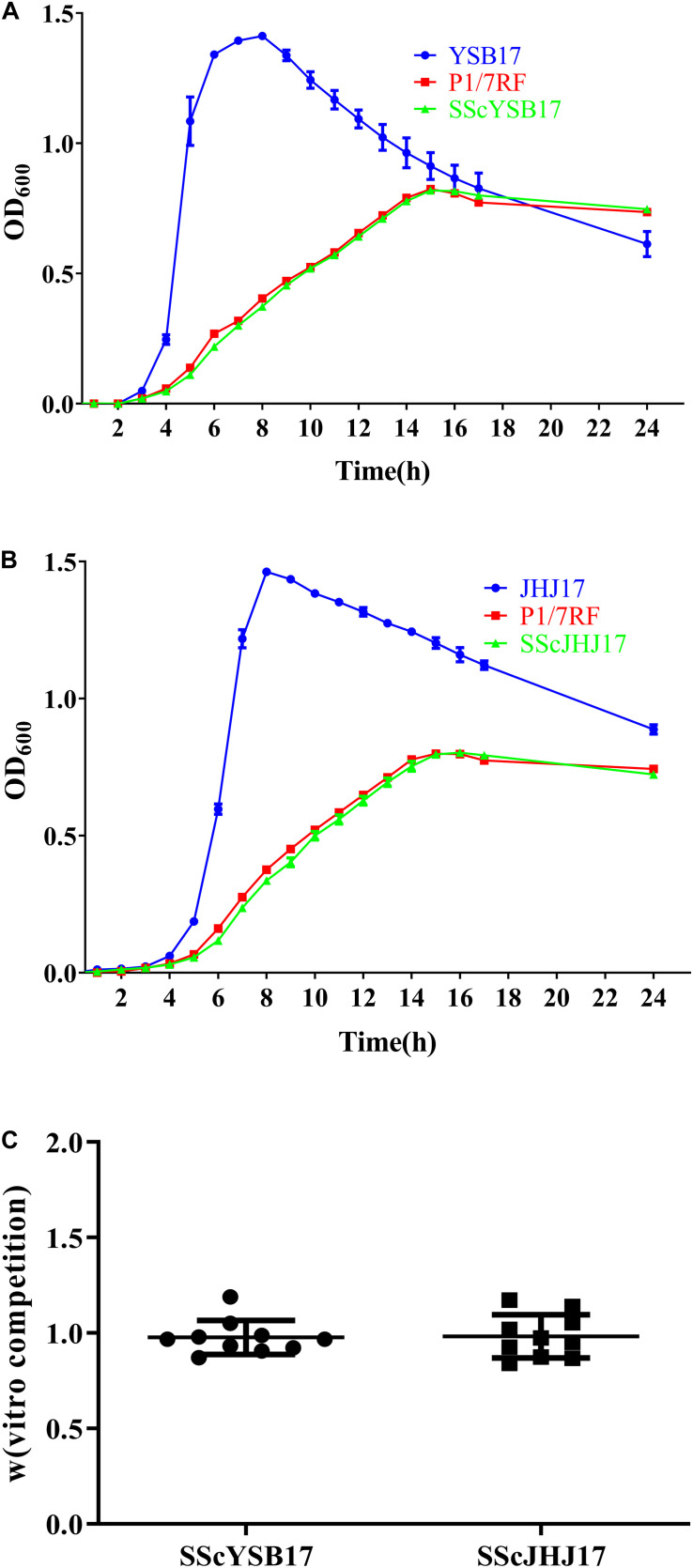
The biological cost of the horizontal acquisition of ICE*Ssu*YSB17_*rplL* or GI*Ssu*JHJ17_*rpsI*. **(A)** Growth curves of the YSB17, P1/7RF, and SScYSB17 under the same conditions *in vitro*. **(B)** Growth curves of the JHJ17, P1/7RF, and SScJHJ17 under the same conditions *in vitro*. **(C)** The relative competitive fitness W of recipient P1/7RF and two transconjugants SScYSB17 and SScJHJ17 strain. The values are represented as mean ± *SD* of 10 independent experiments.

The biological cost of the horizontal acquisition of ICE*Ssu*YSB17_*rplL* or GI*Ssu*JHJ17_*rpsI* was investigated by *in vitro* growth and competition assays. During the *in vitro* growth assays, no significant differences were observed between the recipient *S. suis* P1/7RF and the two transconjugants SScYSB17 and SScJHJ17, suggesting that the acquisition of ICE*Ssu*YSB17_*rplL* (Figure 2A) and GI*Ssu*JHJ17_*rpsI* (Figure 2B) did not affect bacterial growth in THB medium, although both *S. suis* P1/7RF and the transconjugants showed growth delay compared with the donors and the original *S. suis* P1/7 strain.

*In vitro* competition assays showed that the transconjugants SScYSB17 and SScJHJ17 had relative fitness values W of 0.977 ± 0.085 and 0.982 ± 0.108, respectively, when compared with the recipient strain P1/7RF (Figure 2C). These results further suggest that there was no visible fitness cost when recipient strain acquired ICE*Ssu*YSB17_*rplL* or GI*Ssu*JHJ17_*rpsI*.

## Discussion

Macrolide-resistant *Streptococcus pneumoniae*, *S. pyogenes*, and *Streptococcus agalactiae* are 3 of the top 18 drug-resistant threats as declared by the Centers for Disease Control and Prevention (CDC) in the United States in 2013 (CDC, 2013). Previous studies have suggested that *S. suis* is a reservoir of antimicrobial resistance (AMR) genes for other streptococcal pathogens (Palmieri et al., 2011; Huang et al., 2016a). The *erm*(B) gene is the most prevalent determinant conferring resistance to macrolide in streptococci clinical isolates (Chu et al., 2009; Haenni et al., 2018; Ichikawa et al., 2020). However, knowledge about the transfer of *erm*(B) as well as the related MGEs in *S. suis* remains unclear. In this study, we reported the co-transfer of *erm*(B) with other AMR genes among *S. suis* strains mediated by ICEs or GI, which could reveal the reason for the fast spread of macrolide-resistant *S. suis* in recent years in China.

Co-transfer of *erm*(B) and *tet*(O) was confirmed in two strains of *S. suis* serotype 21, which is co-located on ICEs of the ICE*Sa*2603 family. ICE*Sa*2603 family is highly prevalent in major *Streptococcus* species (Davies et al., 2009; Ambroset et al., 2015; Huang et al., 2016b). A variety of resistance genes responsible for resistance to tetracyclines, macrolides, or phenicols have been shown to be transferred inter-strains or inter-species by this family of ICEs (Chen et al., 2007; Palmieri et al., 2012; Marini et al., 2015; Huang et al., 2016b,c; Libante et al., 2019; Pan et al., 2019). Since *erm*(B) and *tet*(O) are located on two different variable regions, namely, HS-2 and I-1, and these two segments showed nearly identical sequence similarity to the corresponding sequences in *S. suis* and other Gram-positive cocci (Supplementary Figure S2), it is reasonable to speculate that ICE*Ssu*YSB17_*rplL* was evolved from acquisition of *erm*(B)-carrying HS-2 and *tet*(O)-carrying I-1 elements through a multi-step process. These results revealed the important role of the acquisition of AMR genes in ICEs diversity and evolution.

Co-transfer of *erm*(B) and *aadE-spw*-like elements was mediated by a novel GI, GI*Ssu*JHJ17_*rpsI*, which is integrated at the *rpsI* site, a conserved hotspot in *Streptococcus* species that was commonly integrated by IMEs and ICEs (Ambroset et al., 2015; Coluzzi et al., 2017; Libante et al., 2019). GIs are usually detected integrated into the 3’-end of the *tRNA* gene. However, two GIs were found in the *rpsI* gene, one carrying the *ant*(9)–*lnu*(C)–*erm*(B) genes (Libante et al., 2019) and another carrying the *aadE*-*lnu*(B)–*lsa*(E)–*spw*-like genes (Huang et al., 2016c). Moreover, GIs integrated into *rpsI* could be mobilized by subverting the relaxase and mating apparatus of a co-resident ICE (Libante et al., 2019). In this study, we also confirmed that GI*Ssu*JHJ17_*rpsI* was able to transfer from a *S. suis* serotype 29 isolate to serotype 2. We speculated that the transfer of GI*Ssu*JHJ17_*rpsI* was mobilized by a *tet*(O)-carrying ICE that harbored a fully functional mobilization module. It needs to be further proven by the inactivation of the *tet*(O)-carrying ICE. Studies have shown that some GIs not only need conjugative elements to promote their own transfer but also influence the transfer or stability of the helper co-resident elements (Guedon et al., 2017).

ICEs could integrate into the chromosome of bacteria and are capable to transfer to a new host uponconjugative transfer (Johnson and Grossman, 2015; Santoro et al., 2018). Functional ICEs were shown to excise from chromosome by site-specific recombination between *att*L and *att*R recombination sites, thus producing a covalently closed circular form of the ICE and a chromosomal excised *att*B site (Puymege et al., 2013). Under normal growth conditions, ICE*Ssu*YSB17_*rplL* and GI*Ssu*JHJ17_*rpsI* are mainly integrated into the chromosome. To check this integration state, we used primer pairs P1 + P2 and P3 + P4 to detect ICE*Ssu*YSB17_*rplL* in the chromosome and primer pairs P5 + P6 and P7 + P8 for GI*Ssu*JHJ17_*rpsI*. However, both MGEs could be excised from the bacterial genome and generated the extrachromosomal circular forms of ICE*Ssu*YSB17_*rplL* and GI*Ssu*JHJ17_*rpsI*, which identified the product by primers of P2 + P3 and P6 + P7, respectively. Furthermore, the empty *rplL att*B or *rpsI att*B’ sites were detected by primers P1 + P4 and P5 + P8, respectively (Supplementary Table S4). This suggests that ICE*Ssu*YSB17_*rplL* and GI*Ssu*JHJ17_*rpsI* are functional and thus have the potential to be transferred. Previous studies have shown that excision of ICEs could be induced under environmental stress, including antimicrobials, such as ciprofloxacin and tetracycline (Beaber et al., 2004; Liu et al., 2017; Scornec et al., 2017). Considering the extensive use of antimicrobials in livestock and poultry, it is of great significance to evaluate the selection stress, especially antimicrobials, which are involved in inducing the excision and thereafter the transfer of the ICEs/GIs.

The acquisition of MGEs was thought to impose an immediate biological cost (Leon-Sampedro et al., 2016). However, the acquisition of ICE*Ssu*YSB17_*rplL* or GI*Ssu*JHJ17_*rpsI* in this study showed negligible fitness cost (Figure 2), which is consistent with our previous study (Huang et al., 2016b). In addition, the AMR-carrying ICEs or GIs enhance their survival under the corresponding antimicrobials. Those may explain the observation that the AMR-carrying ICE*Ssu*YSB17_*rplL*, GI*Ssu*JHJ17_*rpsI*, and similar ICEs are widely distributed in streptococci (Ambroset et al., 2015; Libante et al., 2019).

In summary, we identified three *erm*(B)-carrying transferable elements, including two *erm*(B)- and *tet*(O)-harboring ICEs of the ICE*Sa*2603 family and a novel *erm*(B)-carrying GI, which can be transferred between *S. suis* of different serotypes. The intraspecific transfer of *erm*(B)-carrying MGEs among different serotypes of *S. suis* strains might have contributed to the worldwide spread of macrolide resistance. This reinforces the need for strategies that inhibit the horizontal gene transfer of AMR-carrying MGEs.

## Data Availability Statement

The datasets presented in this study can be found in online repositories. The names of the repository/repositories and accession number(s) can be found below: (Repository: Genbank) (Accessions: BankIt2297772 Seq1 MN876247; BankIt2297772 Seq2 MN876248).

## Author Contributions

LC, JH, and LW developed the concept and designed the experiments. LC, JS, XD, and XW performed the experiments and collected the data. LC, XH, and YH conducted all bioinformatics analyses. LC, JH, MS, and LW prepared the manuscript. All authors have contributed to, seen, and approved the manuscript.

## Conflict of Interest

The authors declare that the research was conducted in the absence of any commercial or financial relationships that could be construed as a potential conflict of interest.

## Footnotes

1 faculty.washington.edu/marilynr/

2 http://rast.nmpdr.org

3 https://db-mml.sjtu.edu.cn/ICEfinder/ICEfinder.html
