# Supplementary material for: Horizontal Transfer of Different erm(B)-Carrying Mobile Elements Among Streptococcus suis Strains With Different Serotypes
Source: Front Microbiol. 2021 Mar 26;12:628740. doi: 10.3389/fmicb.2021.628740 (PMC8032901; doi:10.3389/fmicb.2021.628740)
Supplement: Supplementary file 1 [file Table_1.DOCX]

Supplementary Material

**Supplementary Table 1.** Primers used in this study

| **Target genes** | | **Sequence(5'–3')** | **Product length(bp）** | **Annealing**  **temperature(℃)** | **Source or Reference** |
| --- | --- | --- | --- | --- | --- |
|  | **For detection of resistance genes** | | | | |
| *erm*(B)-F | | TCATCTATTCAACTTATCGTC | 359 | 50 | (Palmieri et al., 2012) |
| *erm*(B)-R | | CTGTGGTATGGCGGGTAAG |  |  |  |
| *tet*(O)-F | | AACTTAGGCATTCTGGCTCAC | 519 | 60 | (Tenover et al., 1987) |
| *tet*(O)-R | | TCCCACTGTTCCATATCGTCA |  |  |  |
|  | **For verification of conjugants** | | | | |
| *CPS2I*-F | | TTCGTATTAACTTACTTGGCGT | 363 | 58 | (Liu et al., 2013) |
| *CPS2I*-R | | TAAATCCCCATATGCCAAATCC |  |  |  |
| *virB4*-F | | TGCCCGCTCCAGAAGTCCCTAAAA | 339 | 55 | This study |
| *virB4*-R | | TTGGCGTGCTTGCGGATACTGA |  |  |  |
| *Int_tyr_-F* | | TCAGCATGCCCCATTCGTCTTG | 126 | 55 | This study |
| *Int_tyr_-R* | | TAAAGCGCAGCAAGTAGGAGTGCC |  |  |  |
|  | | **For quantification of the residual DNA** |  |  |  |
| *virB4*-q-F | | TACTTGGTGGAGAGGAGCGA | 134 | 60 | This study |
| *virB4*-q-R | | GGAAAGGTAGGTTGGGGCAA |  |  |  |
|  | **For probe in southern blotting** | | | | |
| *erm*(B)-p-F | | AACGACGAAACTGGCTAA | 413 | 50 | This study |
| *erm*(B)-p-R | | CTGTGGTATGGCGGGTAA |  |  |  |
| *tet*(O)-p-F | | AGGCATTCTGGCTCAC | 276 | 50 | This study |
| *tet*(O)-p-R | | AATAATACTGCTCCGTCTA |  |  |  |
|  | **For detection of** **circular/integrate form of ICE in *S. suis*** | | | | |
| P1 (hyd) | | GCGGTCGATAGGAACAACC | 748 | 55 | (Huang et al., 2016) |
| P2 (ICE-int) | | AACAAAGACTCCAGCAGGTGA |  |  |  |
| P3 (ICE-repA) | | GCCCCATCCTCATCAATCC | 719 | 55 |  |
| P4 (rplL) | | AAAGTTGGCGTTATCAAAG |  |  |  |
|  | **For detection of circular/integrate form of GI in *S. suis*** | | | | |
| P5 (pyr) | | CCGTCATCGCAACTCTTT | 1012 | 55 | This study |
| P6 (GI-cad) | | ATCTGCCTCCCACCATCT |  |  |  |
| P7 (GI-int) | | CCTAAAGCATTGGCAAGAAC | 902 | 55 |  |
| P8 (rpsI) | | ATGGCACAAGCACAATACAC |  |  |  |

Reference

Huang, J., Liang, Y., Guo, D., Shang, K., Ge, L., Kashif, J., et al. (2016). Comparative Genomic Analysis of the ICE*Sa*2603 Family ICEs and Spread of *erm*(B)- and *tet*(O)-Carrying Transferable 89K-Subtype ICEs in Swine and Bovine Isolates in China. *Front Microbiol* 7**,** 55. doi: 10.3389/fmicb.2016.00055.

Liu, Z., Zheng, H., Gottschalk, M., Bai, X., Lan, R., Ji, S., et al. (2013). Development of multiplex PCR assays for the identification of the 33 serotypes of *Streptococcus suis*. *PLoS One* 8(8)**,** e72070. doi: 10.1371/journal.pone.0072070.

Palmieri, C., Magi, G., Mingoia, M., Bagnarelli, P., Ripa, S., Varaldo, P.E., et al. (2012). Characterization of a *Streptococcus suis* *tet*(O/W/32/O)-carrying element transferable to major streptococcal pathogens. *Antimicrob Agents Chemother* 56(9)**,** 4697-4702. doi: 10.1128/AAC.00629-12.

Tenover, F.C., LeBlanc, D.J., and Elvrum, P. (1987). Cloning and Expression of a Tetracycline Resistance Determinant From Campylobacter Jejuni in *Escherichia Coli*. *Antimicrobial Agents and Chemotherapy* 31(9)**,** 1301-1306. doi: doi: 10.1128/aac.31.9.1301.

**Supplementary Table 2.** Donor strains used in this study

| **Strain** | **Serotype** | **ST** | **Origin** | **Year** | **Geographic** | **Resistance genes** |
| --- | --- | --- | --- | --- | --- | --- |
| HCJ3 | NCL17^a^ | 1075 | Healthy carrier pig | 2016 | Jiangsu, China | *erm*(B), *tet*(O), *aadE, aac(6')-aph(2''), optrA, dfrG* |
| JHB1 | 24 | 1079 | Diseased pig | 2016 | Jiangsu, China | *erm*(B), *tet*(O), *aadE,* *optrA* |
| JHB4 | Non-typeable | - | Diseased pig | 2016 | Jiangsu, China | *erm*(B), *tet*(O) |
| JHJ17 | 29 | 288 | Healthy carrier pig | 2016 | Jiangsu, China | ***erm*(B)**, *tet*(O), ***aadE, spw-like*** |
| YSB17 | 21 | 1095 | Diseased pig | 2016 | Jiangsu, China | ***erm*(B), *tet*(O)** |
| YSJ15 | 21 | 1095 | Healthy carrier pig | 2016 | Jiangsu, China | ***erm*(B), *tet*(O)** |

^a^: NCL17 represent the strain carries a cps locus belonging to novel capsular polysaccharide loci (NCL) type 17.

Genes can be transferred in conjugation assays were shown in bold.

**
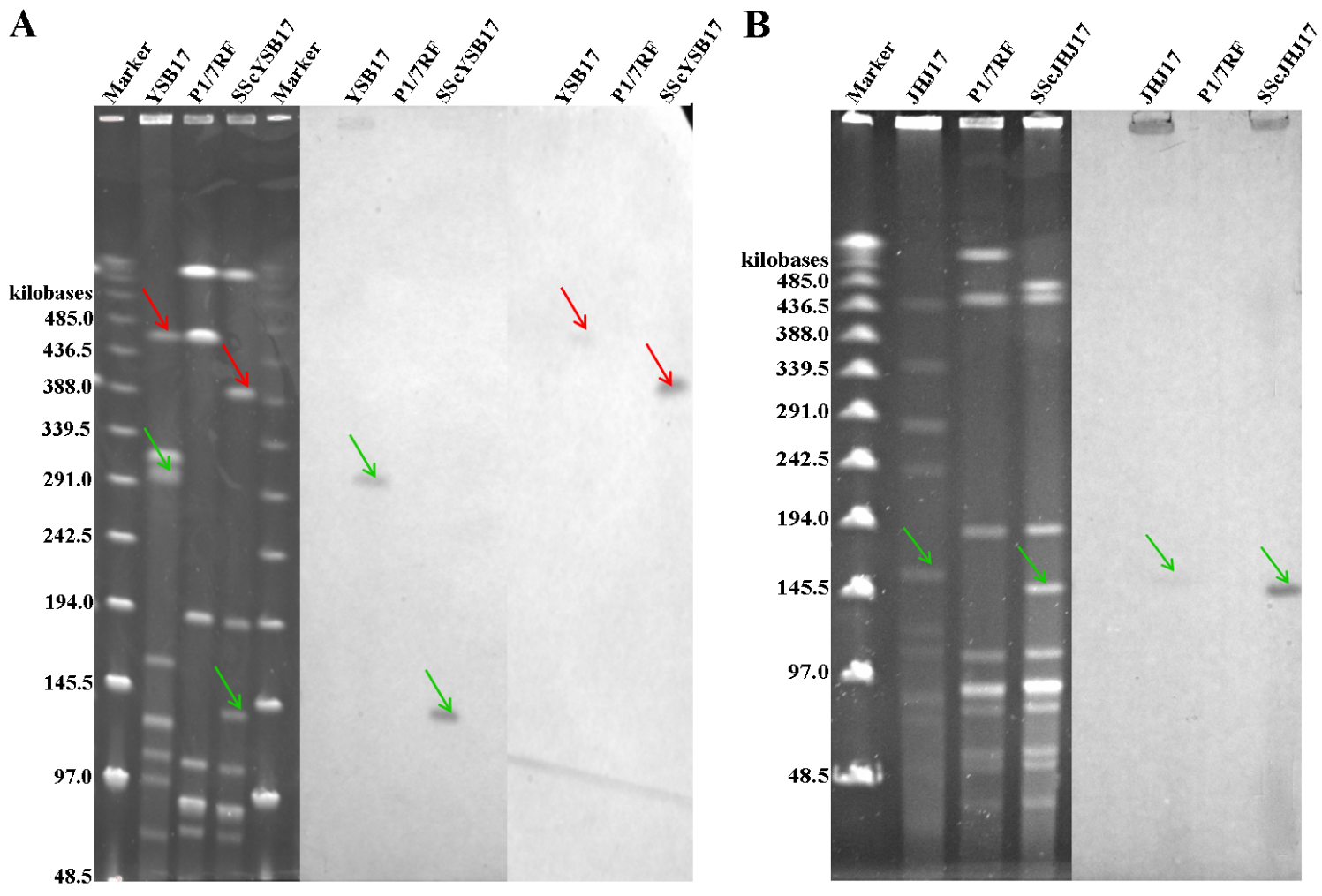
**

**Supplementary Figure 1. PFGE and hybridization studies.** **(A)**, PFGE patterns of *Sma*I-digested genomic DNA (left panel) and Southern-blot hybridization of *erm*(B) (middle panel) and *tet*(O) (right panel) probes of ICE*Ssu*YSB17_*rplL* -carrying *S. suis* isolate and its transconjugant. **(B)**, PFGE patterns of *Sma*I-digested genomic DNA (left panel) and Southern-blot hybridization of *erm*(B) probe (right panel) of GI*Ssu*JHJ17_*rpsI* -carrying *S. suis* isolate and its transconjugant. Lane Marker is Lambda PFG Ladder. Locations of the *erm*(B)- and *tet*(O)-carrying fragment are highlighted by green and red arrows, respectively.

**
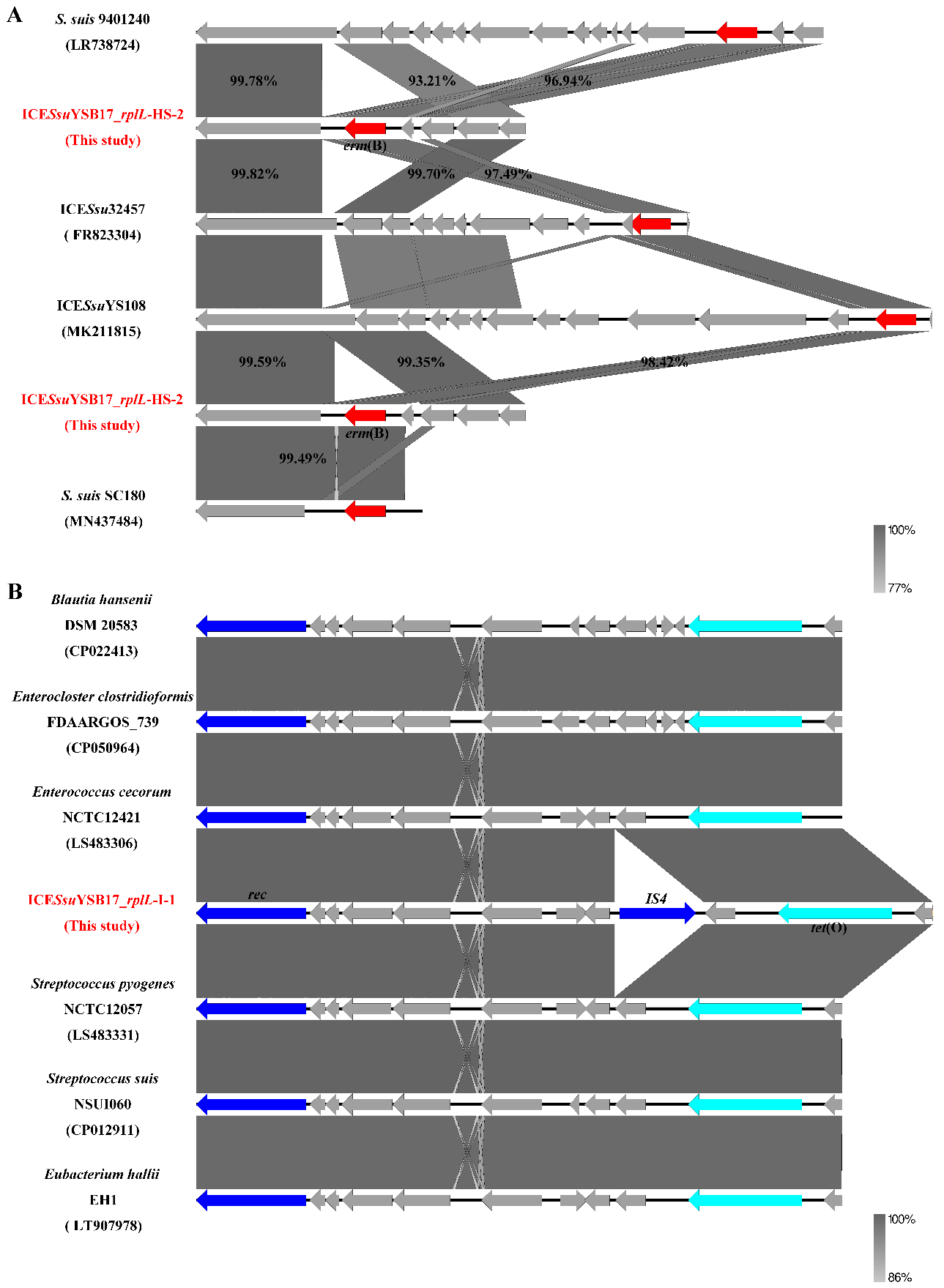
**

**Supplementary Figure 2.** **Genetic features of HS-2 and I-1 from ICE*Ssu*YSB17_*rplL*.** The direction of the arrow indicates the direction of transcription. Homologous regions are shaded in gray. Genes are shown in different colors: *erm*(B) is marked in red, *tet*(O) is labeled in pale blue, recombinase, and transposase genes were highlighted in blue, and other genes are light gray. **(A)**, Comparison of the genetic contexts of *erm*(B) in HS-2 with other bacteria. **(B)**, Comparison of the genetic contexts of *tet*(O) in I-1 with other bacteria.


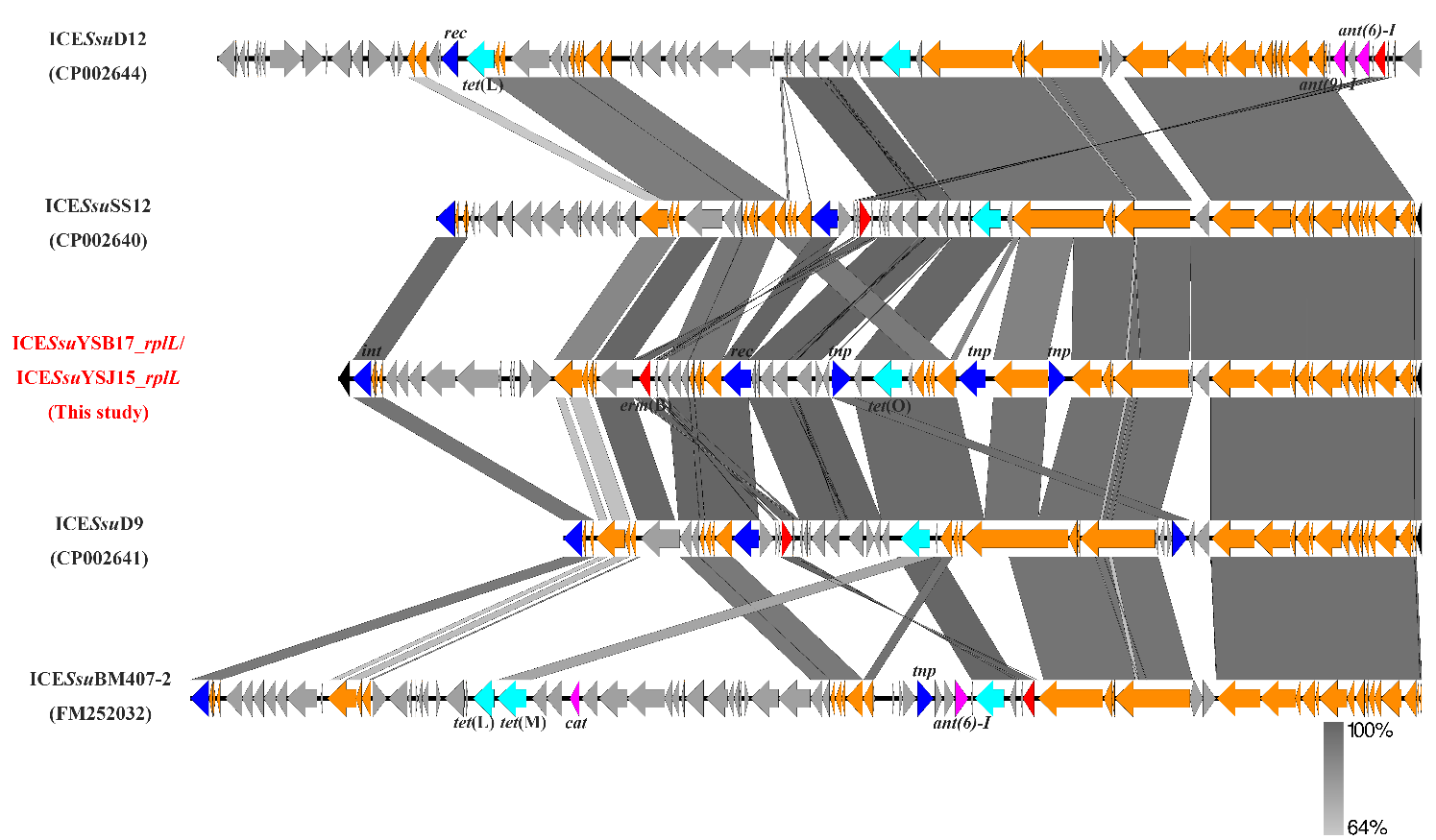


**Supplementary Figure 3.** **Comparison of ICE*Ssu*YSB17_*rplL*/ICE*Ssu*YSJ15_*rplL* with other *erm*(B)- and *tet*(O)-carrying ICEs.** The direction of the arrow indicates the direction of transcription. Homologous regions are shaded in gray. Genes are shown in different colors: the ICE flanking chromosomal genes were shown in black, the 30 conserved core genes of the ICE*Sa*2603 family backbone are orange, variable genes are light gray. Integrase genes were highlighted in blue, *erm*(B) is marked in red, *tet*-resistant genes are in pale blue and other resistant genes are labeled in pink.


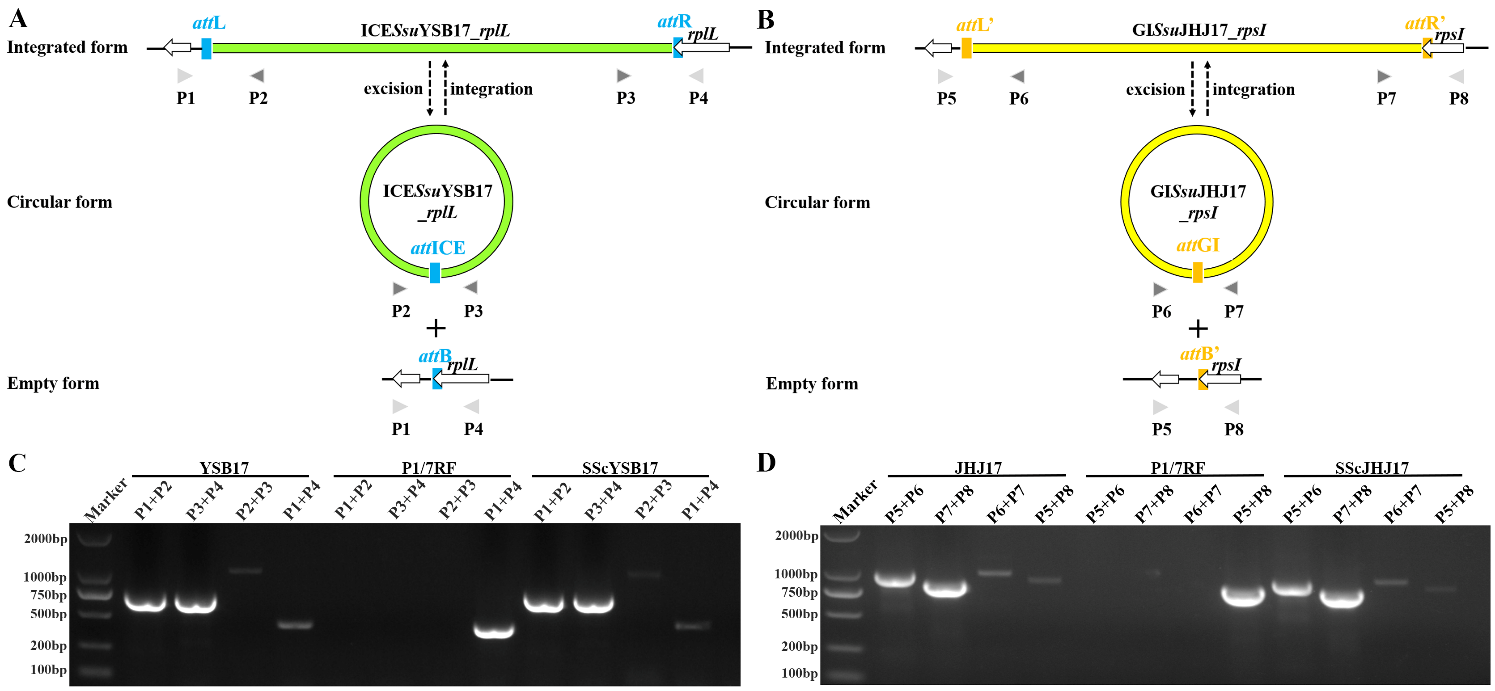


**Supplementary Figure 4.** **The integrated and circular form of ICE*Ssu*YSB17_*rplL* and GI*Ssu*JHJ17_*rpsI*. (A)**, Schematic illustration of ICE*Ssu*YSB17_*rplL* excises from chromosome producing a circular form and a reconstitution of *att*B insertion site. Att sites are represented as blue filled rectangles in ICE*Ssu*YSB17_*rplL*, chromosomal genes as open arrows, ICE*Ssu*YSB17_*rplL* as the green bar. Arrowheads represent PCR primers used for circular form (dark gray) and reconstituted *att*B site (light gray) detection. **(B)**, Schematic illustration of GI*Ssu*JHJ17_*rpsI* excises from chromosome producing a circular form and a reconstitution of *att*B’ insertion site. Att sites are represented as orange filled rectangles in GI*Ssu*JHJ17_*rpsI*, chromosomal genes as open arrows, GI*Ssu*JHJ17_*rpsI* as the yellow bar. Arrowheads represent PCR primers used for circular form (dark gray) and reconstituted *att*B’ site (light gray) detection. **(C)**, PCR products using specific primer pairs by the integrated form and the extrachromosomal circular form of the ICE*Ssu*YSB17_*rplL.* **(D)**, PCR products using specific primer pairs by the integrated form and the extrachromosomal circular form of the GI*Ssu*JHJ17_*rpsI*.
